# Role of Liver Function in the Multiparametric Assessment of Hepatocellular Carcinoma

**DOI:** 10.3390/medicina62010138

**Published:** 2026-01-09

**Authors:** Fabio Melandro, Leonardo Centonze, Ciro Celsa, Simone Famularo, Davide Ghinolfi, Silvia Nardelli, Maria Pallozzi, Ludovico Abenavoli, Fabrizio Romano, Francesca Romana Ponziani, Francesco Paolo Russo, Quirino Lai

**Affiliations:** 1 Department of General and Specialist Surgery, “Sapienza” University of Rome, 00100 Roma, Italy; 2 Department of General Surgery and Transplantation, Azienda Socio-Sanitaria Territoriale Grande Ospedale Metropolitano Niguarda, Piazza Ospedale Maggiore 3, 20162 Milan, Italy; leonardo.centonze@ospedaleniguarda.it; 3 Gastroenterology and Hepatology Unit, Department of Health Promotion, Mother & Child Care, Internal Medicine & Medical Specialties, University of Palermo, 90133 Palermo, Italy; 4 Hepatobiliary Surgery Unit, Fondazione Policlinico Universitario Gemelli IRCCS, 00168 Rome, Italy; 5 Division of Hepatic Surgery and Liver Transplantation, University of Pisa Hospital, 56126 Pisa, Italy; 6 Department of Translational and Precision Medicine, “Sapienza” University of Rome, 00100 Rome, Italy; silvia.nardelli@uniroma1.it; 7 Hepatology Unit, CEMAD Centro Malattie dell’Apparato Digerente, Medicina Interna e Gastroenterologia, Fondazione Policlinico Universitario Gemelli IRCCS, 00168 Rome, Italy; 8 Department of Health Sciences, University of Catanzaro “Magna Graecia”, 88100 Catanzaro, Italy; l.abenavoli@unicz.it; 9 School of Medicine and Surgery, University of Milan-Bicocca, 20900 Monza, Italy; 10 Gastroenterology and Multivisceral Transplant Unit, Azienda Ospedale Università di Padova, 35121 Padua, Italy

**Keywords:** hepatocellular carcinoma, liver function, tace, tare, liver transplantation, immunotherapy

## Abstract

Liver function plays a pivotal role in the management of hepatocellular carcinoma (HCC). Consequently, managing HCC requires a dual focus on both tumour staging and liver function assessment to guide therapeutic decisions. Comprehensive liver function evaluation involves clinical tools such as the Child–Pugh classification and the Model for End-Stage Liver Disease (MELD) score. This is supplemented by newer metrics, including the MELD-Na score, the albumin–bilirubin (ALBI) grade and liver stiffness measurements. These assessments are integral to tailoring treatments, ranging from curative approaches such as surgical resection and liver transplantation to locoregional options (percutaneous ablation, transarterial chemoembolisation and radioembolisation), and systemic therapies. This review explores strategies for balancing the aggressiveness of cancer therapy with the need to preserve hepatic function, particularly in patients with advanced liver dysfunction. A multidisciplinary approach, incorporating expertise from hepatology, oncology, radiology and surgery, is essential for optimising outcomes. Advanced imaging techniques and biochemical markers also improve decision-making and ensure individualised care.

## 1. Introduction

The global burden of primary liver cancer is considerable and growing steadily. According to estimates from 2020, liver cancer is the sixth most commonly diagnosed cancer and the third leading cause of cancer-related deaths [1]. This condition is uniquely complex to manage, not only because of the tumour itself, but also because it frequently arises in the context of chronic liver disease or cirrhosis [2]. Patients with chronic liver disease experience persistent hepatic inflammation, fibrosis and abnormal hepatocyte regeneration. These processes can lead to cirrhosis and favour a sequence of molecular events that culminate in the development of hepatocellular carcinoma (HCC) [3].

Cirrhosis is present in up to 90% of HCC cases and represents a major risk factor for both cancer development and poor prognosis. It is associated with reduced liver function, impaired regenerative capacity and clinically significant portal hypertension (CSPH)—factors that affect prognosis and constrain therapeutic possibilities. Therefore, a comprehensive evaluation of HCC must incorporate not only tumour staging, but also a precise assessment of liver function, as this plays a pivotal role in determining the therapeutic strategy—from curative treatments such as resection and liver transplantation (LT), to bridging or palliative approaches including transarterial chemoembolisation (TACE), transarterial radioembolisation (TARE) and systemic therapies [4] (Table 1).

In recent years, the clinical approach to HCC has transitioned from a rigid, staging-based model to a more nuanced, multiparametric, multidisciplinary process incorporating hepatologists, oncologists, radiologists and surgeons [5]. Central to this evolution is a refined understanding of life expectancy in HCC based on tumour burden, liver function and cancer-related symptoms, as acknowledged in the 2025 BCLC update [6]. Cirrhosis status is a key factor in determining prognosis and is the first consideration in clinical frameworks. In patients with cirrhosis, distinguishing between compensated and decompensated disease is important. In decompensated patients, the prognosis is determined by liver function, whereas in compensated patients, it is determined by tumour burden.

Furthermore, the idea that liver function can be adequately captured by a single score (e.g., Child–Pugh, CP) is increasingly being questioned. More flexible and individualised strategies are now required, especially given the growing complexity of therapeutic options and patient heterogeneity [7]. This new approach is based on the concept of the Multiparametric Therapeutic Hierarchy (MTH), which is a more realistic alternative framework for clinical decision-making in HCC. This concept endorses the prognostic independence of treatment modalities from conventional staging systems and the persistence of a therapeutic efficacy gradient within each BCLC stage, in the following order: LT > resection > ablation > embolisation > systemic therapy.

When curative treatments are initially contraindicated, the goal may shift towards a ‘converse therapeutic hierarchy’—the use of systemic or intra-arterial therapies to modify tumour biology and enable subsequent curative intent (conversion therapy). Modern systemic agents, particularly lenvatinib and immunotherapy, have markedly improved objective response rates, supporting this paradigm shift [8].

Within the MTH model, the multidisciplinary tumour board (MTB) plays a central role in assessing the feasibility of each treatment option, ranked by survival benefit, while retaining the flexibility to personalise care. The selection or exclusion of a given treatment is based on a structured, evidence-based evaluation of key variables, including liver function, patient fitness, tumour complexity, technical feasibility, treatment availability, cost and resource allocation. The process also considers treatment harms and benefits, societal values and preferences and broader dimensions such as equity, feasibility and acceptability [9].

Liver dysfunction is a key factor in the MTH model for HCC, influencing both treatment feasibility and patient prognosis. While all major algorithms incorporate liver function assessment, this is often limited to the CP classification, which does not capture the full complexity of hepatic reserve. In reality, liver function is a dynamic, multidimensional parameter that should be assessed using a combination of biochemical, hemodynamic and structural markers. Tools such as the Model for End-Stage Liver Disease (MELD) and MELD-Sodium (MELD-Na) scores, the albumin–bilirubin (ALBI) grade, the indocyanine green retention test after 15 min (ICG-R15), liver and spleen stiffness and indicators of CSPH—including the hepatic venous pressure gradient (HVPG) and estimation of the future liver remnant volume (FLRV)—provide a more nuanced appraisal of hepatic functional capacity. This integrated assessment is pivotal in predicting postoperative decompensation, determining eligibility for surgical, locoregional or systemic therapies and guiding longitudinal treatment adaptation [8,9].

Furthermore, the aetiology of liver disease (viral, metabolic, alcoholic or autoimmune) affects both the progression of liver dysfunction and the tolerance of therapy, highlighting the importance of an individualised, aetiology-informed evaluation. Within the context of the horizontal therapeutic axis, precise and continuous assessment of liver function ensures balanced decision-making—aligning oncological ambition with hepatic safety, while preserving the potential for future curative strategies [10].

This review explores the central role of liver function within this MTH, examining its prognostic significance, its effect on eligibility for treatment and the most reliable tools for evaluating it in surgical and non-surgical settings. By incorporating liver function into a dynamic, hierarchical and patient-centred algorithm, our aim is to provide a conceptual and practical framework that overcomes the limitations of traditional models and facilitates more effective and personalised treatment planning.

## 2. Liver Transplantation

In HCC, LT is the ultimate curative step within the MTH, as it removes the tumour and replaces diseased liver parenchyma simultaneously. This dual action makes LT the optimal treatment for HCC patients with advanced liver dysfunction, provided tumour biology remains favourable [11]. However, due to the scarcity of donor organs, candidate selection must adhere to the principles of utility, urgency and transplant benefit.

Importantly, severe liver dysfunction is not an absolute contraindication for LT unless it contributes to multi-organ failure. Conversely, patients with HCC and decompensated cirrhosis but without biologically aggressive tumour features derive the greatest benefit from a transplant, as LT offers them both tumour eradication and functional hepatic restoration. For these individuals, other curative options, such as resection or ablation, are usually ruled out due to poor hepatic reserve, which highlights the important role that liver function plays in determining the most suitable treatment option [12].

Current evidence shows that LT achieves excellent long-term outcomes, with five-year overall survival (OS) exceeding 70% in appropriately selected patients, and a survival curve that plateaus after transplantation, indicating a potential cure. Post-transplant outcomes are only minimally influenced by pre-existing liver function. This is why, within the MTH framework, there are no specific hepatic function thresholds that would preclude LT, in contrast to all other therapeutic options. Thus, while impaired liver function may exert some effect on outcomes, its impact is dramatically lower than for alternative therapies and is generally not considered an absolute contraindication to transplantation—except in situations where hepatic failure progresses to multiorgan dysfunction, as observed in acute-on-chronic liver failure. Although several limitations have been highlighted, the MELD score continues to represent the gold standard for pre-transplant disease severity assessment and liver allocation, where its prognostic performance is well established. Therefore, improving pre-transplant optimisation and early postoperative management is essential to enhance early survival rates [13].

In some countries, such as Japan, LT eligibility continues to reflect the importance of liver dysfunction: public insurance coverage currently extends only to patients with decompensated liver disease (CP class B or C) who meet the established transplant criteria. Recent policy updates, including lowering the CP threshold from ≥10 to ≥7 and increasing the age limit for donors, aim to make LT more accessible for patients with HCC and significant hepatic impairment. In this context, liver dysfunction is a decisive factor in the hierarchical framework, determining both the indication for and prioritisation of LT. This emphasises that balancing tumour biology and hepatic reserve is key to maximising therapeutic success [14].

All of the above demonstrates that the liver transplant procedure is capable of effectively tackling the issue of impaired liver function.

## 3. Hepatic Resection

Recent refinements in patient selection and preoperative risk stratification, coupled with increased proficiency in minimally invasive liver resection, have contributed to a paradigm shift in patient eligibility for curative surgery. This procedure can now be considered a safe option, even in cases of advanced cirrhosis and CSPH.

Minimally invasive approaches (laparoscopic or robotic) offer several advantages in terms of functional recovery and perioperative outcomes, providing better outcomes and a shorter hospital stay than open surgery [15,16,17].

In recent years, research has focused on investigating possible relationships between minimally invasive surgery and the potential extension of surgical options for cirrhotic patients. Minimally invasive approaches could enhance the potential for safe curative surgery, even in patients with CSPH, who are usually not considered optimal candidates for resection [18,19]. In particular, two prospective multicentre studies, highlighted that, in well-selected patients, a laparoscopic approach could be an independent predictor of achieving a favourable outcome in this higher-risk population, enabling a safer extension of surgical indications for this group of patients [20,21].

The CP classification system continues to be widely used in decision-making regarding liver resection. Patients in CP class B have a significantly poorer prognosis than those in CP class A concerning both short- and long-term outcomes [22].

A recent retrospective study by Kou et al. identified key determinants of early mortality within two years after resection, including CP class B, advanced BCLC stage and a high Tumour Burden Score [23].

Wang et al. [24] reported that the ALBI grade more accurately predicts post-hepatectomy liver failure (PHLF) than the CP grade. The ALBI score is divided into grades 1, 2 and 3: grades 1 and 2 seem to recognise two different subgroups of patients with CP class A, enabling the risk of these patients to be stratified more effectively than with the CP system.

MELD score has historically been recognised as a predictor of PHFL. Teh et al. [25] found an increased risk of perioperative mortality in patients with a MELD score greater than 9, recommending that minor or major resection only be performed in patients with a score of 8 or below.

Kokudo et al. [26] developed a grading system incorporating two variables: serum albumin level and ICG-R15. This system was called “ALICE grade scale” (albumin–indocyanine green evaluation). A score of 3 was associated with a poor prognosis and a high risk of PHFL. Furthermore, major liver resection among grade 2b patients was found to be associated with significantly higher morbidity and mortality rates.

In addition to blood chemistry tests, clinical imaging based on elastography, scintigraphy and magnetic resonance imaging has been used to assess liver function prior to resection [27].

Measurement of liver and splenic stiffness has been reported to be useful for predicting PHFL; however, this technique has several limitations relating to operator expertise and patient features (e.g., cirrhosis, ascites and body fat).

Magnetic resonance (MR) enhancement using a hepatocyte-specific contrast agent (Gd-EOB-DTPA) has been reported to provide liver tumour features as well as combined anatomical and quantitative liver functional information [28]. The dynamics of the hepatobiliary-specific contrast agent lead to different intensity patterns on MRI scans, making them a kind of indirect sign of hepatocyte function. In recent years, radiomics analysis has enabled precise estimation of signal intensity, demonstrating optimal performance in discriminating between patients with different CP classes (AUC = 0.75, 95% CI: 0.66–0.83) [29]. Furthermore, hepatocellular uptake has been demonstrated to be an effective predictor of PHLF (AUC = 0.84; 95% CI: 0.71–0.92) [30]. Although these techniques are very promising, they are still in the preclinical phase, and there are no reports about their implementation in real-world clinical practice.

Optimising the future liver remnant (FLR) is pivotal to improving postoperative outcomes in HR. The minimal FLR should be approximately 20% of the original hepatic volume in patients with a normal liver, 30% in patients with liver injury (e.g., chemotherapy-induced injury or steatosis) and 40% in patients with cirrhosis [31]. FLR volume can be estimated using cross-sectional imaging (CT, MRI or Tc-99m-GSA scintigraphy) with three-dimensional reconstruction. In addition to FLR volume, it is also important to estimate FLR function [32].

The ICG-R15 test is one of the most commonly used tests to describe liver function and predict liver failure. The first report of a surgical decision-making algorithm based on ICG-R15 appeared in the literature in 1993, by Makuuchi et al. [33]. This algorithm has been shown to reduce hepatectomy-related morbidity and mortality, particularly during the early stages of liver surgery. Since then, this data has been widely used to assess liver function before and after resections. The algorithm represents a better and more effective set of decision-making criteria due to its limitations alone.

The role of HVPG measurement in risk stratification before liver resection remains a topic of ongoing debate. An elevated HVPG (>10 mmHg) indicates CSPH and is linked to a higher risk of complications and reduced survival. However, Cucchetti et al., in a cohort of 70 hepatic resections, reported that approximately one-quarter of patients enrolled experienced a normal and uneventful postoperative course despite having a pressure gradient > 10 mmHg [34].

Wang et al. [35] developed a nomogram to predict PHLF, by integrating preoperative and intraoperative parameters. Seven independent predictors of PHLF were identified: liver cirrhosis; total bilirubin > 34.2 µmol/L; prolonged prothrombin time > 14 s; ALBI score > −2.39; Fibrosis-4 index > 2.67; presence of ascites; and intraoperative blood loss (>2000 mL).

Of all the predictors, intraoperative blood loss was found to be the strongest determinant of PHLF risk. By combining liver function, fibrosis and perioperative factors, this integrative model outperformed traditional liver function scores (e.g., CP, MELD), offering a more comprehensive and accurate risk stratification tool.

## 4. Percutaneous Ablation

Percutaneous ablation (PA) has emerged as a potentially curative treatment for early-stage HCC, particularly for nodules smaller than 3 cm in patients with preserved liver function (CP class A) and a good performance status (performance status 0). Techniques such as radiofrequency (RF) and microwave (MW) ablation are becoming increasingly popular due to their minimally invasive nature and effectiveness.

MW offers several advantages over RF, notably the ability to achieve higher temperatures (>100 °C), resulting in larger and faster ablation zones. It is also less susceptible to the heat sink effect caused by blood flow, which can hinder the effectiveness of RF ablation. Consequently, MW ablation may improve local tumour control, particularly for tumours located near major blood vessels. PA was initially intended for small tumours, but nowadays its application has expanded to include larger nodules (>3 cm) through various technologies and combined approaches, such as intra-arterial therapies and immunotherapy [36].

Recent advancements in imaging techniques, such as real-time computerised fusion, have significantly improved the precision and safety of ablation procedures. Enhanced imaging enables more precise applicator positioning and energy delivery, with the aim of achieving complete tumour coverage while protecting critical structures. Immediate or short-term evaluation criteria for assessing ablation efficacy are crucial for minimising local recurrence rates [37].

Experience indicates that liver functional reserve is a critical determinant of prognosis after PA for HCC, often outweighing tumour factors. Therefore, an accurate assessment of liver function at the time of HCC diagnosis is essential [38].

Critical indicators of liver function include serum albumin levels, platelet counts and liver enzyme levels, such as aspartate transaminase (AST), alanine transaminase (ALT) and alkaline phosphatase [39,40]. Low serum albumin levels are associated with poorer liver functional reserve and worse OS outcomes, as demonstrated in studies by Xu et al. [41] and Toshikuni et al. [42]. Furthermore, lower pretreatment serum albumin/alkaline phosphatase ratios have been associated with poorer OS and RFS [43].

The clinical application of the ALBI grade was tested in a cohort of 499 patients treated with RF, showing significantly better OS rates (77.9% to 88.5%) in patients classified as ALBI grade I, compared to grades II/III (38.6% to 73.8%) [44].

## 5. Transarterial Chemoembolisation

TACE is the standard treatment for patients with compensated HCC who are not eligible for surgical resection or ablation, and who do not have macrovascular invasion and/or extrahepatic disease [45].

The efficacy and safety of TACE are inextricably linked to the patient’s underlying liver function. Patients with CP score ≥ 8, serum bilirubin levels higher than 2 mg/dL and the presence of clinically relevant ascites requiring diuretic treatment are at significantly increased risk of further deterioration of liver function after TACE [46].

Serum albumin plays a pivotal role as a surrogate marker of hepatic synthetic function and has consistently emerged as a strong predictor of outcomes following TACE. Baseline hypoalbuminemia reflects impaired liver reserve and is associated with an increased risk of post-procedural hepatic decompensation, reduced tolerance to repeated TACE sessions and poorer OS. Several studies have demonstrated that lower pre-TACE albumin levels correlate with higher rates of liver function deterioration and treatment discontinuation after TACE: recently, a combination of serum albumin ≤ 3.8 g/dL, PT ≤ 80% and largest tumour diameter ≥ 3.8 cm was reported as a predictor of immediate deterioration of the CP classification from A to B [47].

ALBI score has also demonstrated good prognostic ability in patients undergoing TACE, although it was lower than that of other prognostic tools, which also included tumour-related features. Several studies have shown that ALBI grade is a better predictor of OS and risk of post-procedural complications than the CP and MELD scores [48,49,50,51]. Furthermore, a higher pretreatment ALBI grade III has been found to be an independent predictor of acute-on-chronic liver failure 90 days after the procedure [52].

Compared with the CP score and ALBI, platelet–albumin–bilirubin (PALBI) score showed a better predictive effect in patients with stage C HCC with cirrhosis, treated with c-TACE, as reported [53].

In addition to assessing liver function prior to TACE, careful evaluation and monitoring of changes in liver function parameters is also necessary. It is well known that acute liver injury can occur within the first 30 days after TACE [54]. Although liver function test impairment could be reversible in some patients after TACE, persistent impairment can adversely affect prognosis and limit the effective delivery of further TACE procedures or shift to systemic therapy.

The relevance of changes in bilirubin levels after TACE has also been assessed in a prospective study of 84 Italian patients. This study showed that bilirubin levels, both at baseline and when treated as a time-varying covariate in a time-dependent Cox model, were independently associated with OS [55].

The occurrence of clinical liver decompensation after TACE limits the delivery of subsequent therapies and significantly reduces OS. Furthermore, the lack of systematic and reproducible reporting of clinical liver decompensation in studies evaluating TACE could explain the poor correlation between radiology-based intermediate endpoints, such as time-to-progression or progression-free survival (PFS), and OS [56,57,58].

## 6. Transarterial Radioembolisation

TARE is an advanced interventional procedure used to treat HCC, particularly as a bridge to transplant or resection, or as an alternative to TACE in intermediate stages, and as an alternative to systemic therapy in advanced stages (BCLC groups B and C, respectively) [59]. According to the recent update of the BCLC criteria, TARE may also be considered for BCLC stage 0 patients as an alternative to percutaneous ablation with curative intent, particularly for elderly patients for whom surgery is contraindicated or for patients with challenging nodules to treat with other techniques [60].

The subgroup of patients who will benefit from TARE remains to be clearly defined (moderate evidence). Patients in whom TARE may be considered include those with large solitary tumours, and those with tumours associated with local macrovascular tumour invasion, for whom tolerance to systemic therapy is, or is likely to be, a concern (evidence low; recommendation weak) [61]. Additionally, TARE can be a valuable tool in downstaging tumours, enabling patients with initially unresectable HCC to become eligible for surgical resection or transplantation. Outcomes are influenced by liver functionality and the extent of disease at the time of treatment. Patients with well-preserved liver function tend to respond better and experience fewer complications than those with significant hepatic impairment [62].

TARE uses microspheres loaded with a radioactive isotope, typically yttrium-90 (Y-90), which emits beta radiation to destroy cancer cells. The radiolabelled particles are injected through the hepatic artery and become trapped at the precapillary level, where they emit lethal internal radiation. This mechanism limits exposure to the surrounding normal liver tissue, thereby enabling a higher dose to be delivered than with external beam radiotherapy [63,64]. As the hepatic artery primarily supplies the tumour, most of the radiation is concentrated in the tumour, sparing the surrounding normal liver tissue. This targeted delivery helps achieve localised tumour control with potentially fewer systemic side effects than other therapies [65].

Historically, dosimetry recommendations targeted an average absorbed dose of 120 Gy to the perfused liver for lobar treatment, or greater than 190 Gy to the tumour for ablative radiation in no more than two Couinaud liver segments, i.e., radiation segmentectomy. However, using these recommendations, no distinction was made between tissue-absorbed dose (TAD) and normal tissue-absorbed dose (NTAD) [66]. However, recent prospective and retrospective studies have demonstrated improved outcomes based on personalised dosimetry techniques utilised in both single- and multi-compartment dosimetry. In single-compartment dosimetry, most patients achieve a pathological response if ablative radiation is delivered to a limited perfused volume, with an absorbed dose exceeding 400 Gy [67,68,69].

The liver’s functional reserve determines its ability to tolerate radiation-induced damage and its capacity for recovery after treatment. Therefore, a comprehensive evaluation of liver function is essential before considering TARE. Commonly used tools for this evaluation include the CP classification and the MELD score [70].

In a retrospective review of 102 TARE patients by Kim et al. [71], a low MELD score was found to be an independent predictor of a greater likelihood of a complete imaging response, as well as smaller tumour size, post TARE in patients with HCC.

The ALBI score has also been shown to be beneficial in assessing liver function prior to TARE. Patients with lower ALBI scores (ALBI 1 vs. 2/3) have a lower incidence of radiation-induced liver disease (RILD) (3.4% vs. 16%), and better OS (26.4 months vs. 17.3 months and 8.1 months) after treatment [72]. Quantitative measures, such as hepatic arterial perfusion and tumour-to-liver dose ratio calculations, help optimise the dose of radioactive microspheres and minimise risks to healthy liver tissue. Imaging-guided planning ensures that TARE is administered safely by avoiding excessive radiation to non-tumour liver regions and critical structures such as the gastrointestinal tract [73,74].

In conclusion, TARE plays a significant role in managing HCC, particularly in patients with compromised liver function who are not suitable candidates for surgery.

The interplay between liver functionality and TARE is crucial, as the liver’s capacity to handle radiation-induced damage directly affects the safety and efficacy of the treatment.

## 7. Systemic Therapy

Systemic therapy is the primary treatment for advanced, unresectable HCC. Between 2008 and 2020, only the tyrosine kinase inhibitors (TKIs) sorafenib and lenvatinib demonstrated significant improvements in OS and PFS for HCC patients [75,76]. In 2020, the IMbrave150 trial marked a breakthrough, by demonstrating that the combination of atezolizumab (an immune checkpoint inhibitor (ICI) targeting programmed death ligand 1 (PD-L1)) and bevacizumab (an anti-angiogenic agent) was superior to sorafenib in terms of safety and efficacy. This led to the combination being approved by the U.S. Food and Drug Administration (FDA) as a first-line therapy for HCC [77]. Subsequently, the HIMALAYA trial, which tested a single dose of tremelimumab (an anti-CTLA-4 agent) alongside four-weekly durvalumab (an anti-PD-L1 agent) infusions, also showed improved OS compared to sorafenib [78,79].

Despite these promising advances, the effectiveness of systemic therapy is greatly affected by baseline liver function.

New drugs have been tested in clinical trials with restrictive criteria, and only in patients with well-preserved liver function. From a clinical trial and regulatory standpoint, the exclusive inclusion of CP class A patients has been primarily driven by the need to minimise confounding factors and ensure internal validity of efficacy endpoints. In CP class B or C patients, survival is strongly influenced by cirrhosis-related complications rather than tumour progression [80].

Real-world data provide a more accurate picture, analysing data from patients with decompensated liver disease.

Kudo et al. reported that nivolumab shows clinical activity and an acceptable safety profile in patients with CP class B status who have mild-to-moderate impairment of liver function. Notably, 76% of CP class B patients in this study were classified as B7 [81].

A study conducted on the GIDEON registry, which included CP classes A and B7 patients with advanced HCC treated with sorafenib, reported similar incidences of adverse events for CP classes A and B7, although CP class B patients experienced higher rates of permanent treatment discontinuation and greater severity [82].

In a large multicentre retrospective study conducted by D’Alessio et al. [83] on CP class B patients not included in the IMbrave150 trial, despite a significant difference in median OS between CP-A and CP-B patients (16.8 months vs. 6.7 months), the objective response rate (ORR) was comparable across CP classes (26% vs. 21%), suggesting efficacy despite pre-existing liver compensation.

Finally, a large meta-analysis involving 699 CP class B patients with advanced HCC who were treated with ICI [84] confirmed that mild liver dysfunction was associated with poorer OS and ORR but did not significantly affect the incidence of treatment-related adverse events. Interestingly, in these studies, CP class B7 patients exhibited a survival rate closer to that of CP class A patients than to CP class B8 or B9 patients.

Also in the setting of systemic therapy, the ALBI score has demonstrated adequate and clinically meaningful prognostic performance. Multiple retrospective and post hoc analyses have shown that ALBI correlates with OS, treatment-related toxicity and preservation of liver function across different systemic agents, including multikinase inhibitors and ICI [85].

Importantly, ALBI allows further prognostic stratification within CP class A and B patients, identifying subgroups with heterogeneous outcomes and treatment tolerability.

Of note, in a study comparing prognosis between patients with CP class A and B who underwent Atezolizumab plus bevacizumab, modified ALBI score (mALBI) 1 and 2a classes obtained favorable prognosis, whereas patients classified as CP-B, whose mALBI grade is typically 2b or 3, might experience a low level of therapeutic efficacy [86].

Rimini et al. assessed the prognostic role of ALBI during ICI therapy in real-world patients, categorising them based on IMbrave150 eligibility. Patients who were ineligible for the trial but were classified as ALBI grade 1 showed significantly better OS than patients classified as ALBI grade 2 (16.7 vs. 5.9 months), which highlights the relevance of ALBI in evaluating immunotherapy-eligible patients with mild liver impairment [87].

ALBI score may also offer prognostic insights: patients with ALBI grades 2 or 3 who did not experience a reduction in AFP levels within three weeks of ICI therapy exhibited poorer OS [88].

Accumulating evidence indicates that clinically overt hepatic decompensation exerts a greater impact on prognosis in patients with HCC than tumour progression itself. This concept has gained particular relevance in the era of highly effective immunotherapy (Table 2), which has expanded therapeutic opportunities even for patients with impaired hepatic function, thereby underscoring the critical importance of preserving liver reserve throughout the disease course.

ALBI has also been recognised as a predictor of liver decompensation following treatment [89]. Functional impairment during follow-up significantly impacts prognosis in both early- and advanced-stage HCC. In this context, treating the underlying causes (e.g., antiviral treatment for patients with HBV and HCV cirrhosis) may improve OS and reduce the risk of decompensation [90].

A recent multicentre retrospective study demonstrated that a low baseline platelet count is an independent predictor of liver function deterioration at the time of disease progression in patients with unresectable HCC, treated with first-line atezolizumab plus bevacizumab. Among CP class A patients, those with lower platelet levels were significantly more likely to progress to CP class B at progression, potentially limiting access to subsequent lines of therapy. These findings highlight platelet count as a simple surrogate marker of hepatic reserve and fibrosis severity, reinforcing the importance of liver-centred patient selection and monitoring during immunotherapy-based treatment strategies [91].

In patients with CSPH with either oesophageal or gastric varices at high risk of bleeding (small varices with red signs or any medium/large varices, gastric varices of any size), an adequate primary/secondary prophylaxis should be put in place [80].

It is generally recommended that variceal bleeding risk is detected in candidates for systemic therapy, especially those receiving atezolizumab plus bevacizumab. Furthermore, a history of variceal bleeding is the main predictor of new bleeding episodes during systemic therapy [92]. Assessment of CSPH before starting systemic therapy remains non-standardised. In 2023, Piscaglia et al., following Baveno VII criteria, suggested that oesophagogastroduodenoscopy (EGD) screening could be avoided if liver stiffness is <15 kPa, platelet count is >150,000/µL and there is no evidence of macrovascular invasion, splenomegaly, portosystemic collaterals or thrombosis; otherwise, EGD should be performed [93].

Portal vein thrombosis (PVT) is another unfavourable prognostic factor for HCC. The efficacy of immunotherapy (atezolizumab/bevacizumab and lenvatinib) has been demonstrated in patients with PVT in terms of both hepatic function impairment and therapeutic response [94,95].

In conclusion, systemic therapies are associated with an increased risk of mortality in patients experiencing hepatic decompensation. These findings suggest the need for more intensive monitoring and optimisation of treatments to prevent decompensation in high-risk patients [96].

## 8. Conclusions and Future Directions

The decision-making process for HCC should consider baseline liver function. Although several scoring systems are currently used in clinical practice, including the CP score, MELD, ALBI and ICG-R15 systems, each of these tools has specific limitations that may affect their clinical applicability in certain contexts. Although the CP score is widely used, it relies on semi-quantitative variables (ascites and encephalopathy) and lacks the ability to stratify patients within the same class with sufficient granularity. The MELD and MELD-Na scores, which were originally designed to predict short-term mortality in patients with cirrhosis awaiting LT, may not fully capture liver functional reserve in patients who are not candidates for LT or who are undergoing surgical or locoregional therapies. Despite its simplicity and objectivity, the ALBI score does not account for CSPH or synthetic dysfunction beyond bilirubin and albumin levels.

Furthermore, these scoring systems often behave differently depending on the treatment modality, tumour burden and the underlying aetiology of the liver disease—e.g., viral versus metabolic cirrhosis. Their predictive power for treatment-related complications or survival outcomes is not consistent in different therapeutic contexts. Furthermore, most of these models only provide a static snapshot of liver function and do not incorporate dynamic changes over time, which can be crucial in longitudinal treatment planning or restaging.

Dynamic functional tests such as ICG-R15 and imaging-based biomarkers—including liver stiffness measurement, hepatobiliary contrast-enhanced MRI and quantitative scintigraphy—further contribute to a more physiologically accurate estimation of hepatic reserve. These tools are especially valuable in surgical and interventional settings, where they help predict post-hepatectomy liver failure, radiation-induced liver disease or treatment-related toxicity. Although not yet universally implemented, their integration into multidisciplinary decision-making frameworks represents a promising step toward precision hepatology.

Crucially, improved liver function assessment does not merely stratify risk but can indirectly affect liver function itself by guiding less harmful treatment choices, optimising timing and avoiding overtreatment. By selecting therapies aligned with hepatic functional capacity, clinicians may reduce the incidence of treatment-induced liver injury, preserve eligibility for subsequent therapeutic lines and ultimately improve survival outcomes. This paradigm underscores the shift from a tumour-centric to a liver-centred approach in HCC management [97].

Within the framework of the MTH, liver function assessment tools serve as dynamic modulators rather than rigid gatekeepers, enabling tailored treatment escalation or de-escalation while maintaining oncological efficacy and hepatic safety. Future research should focus on validating integrated models that combine biochemical, clinical and imaging-based parameters, as well as on defining optimal timing for reassessment, to fully exploit the potential of these tools in improving patient outcomes.

To prevent hepatic decompensation, standardised and disease-specific management protocols are needed for chronic viral hepatitis and nonalcoholic fatty liver disease (NAFLD), now known as Metabolic Dysfunction-Associated Steatotic Liver Disease (MASLD), including the structured use of emerging therapies such as resmetirom and GLP-li1 receptor agonists [98].

Prospective studies should define optimal timing for liver function reassessment, for instance within 4–6 weeks prior to locoregional treatments such as TACE or thermal ablation, and at predefined intervals after treatment (e.g., 4–8 weeks), to capture early functional deterioration. Furthermore, such studies should aim to validate alternative approaches for predicting portal hypertension progression and liver function decline, integrating dynamic clinical, biochemical and imaging-based parameters beyond static baseline scores.

Future efforts should focus on integrating precision medicine and developing novel biomarkers that integrate biochemical, radiological and clinical data into composite models to more accurately guide therapeutic decision-making, particularly within the framework of multiparametric and hierarchical treatment selection in HCC.

## Figures and Tables

**Table 1:** Role of liver function across the therapeutic hierarchy of hepatocellular carcinoma.

HCC Treatment	Liver Function Indices Used	Outcomes Influenced
Liver Transplantation	MELD, MELD-Na, Child–Pugh, presence of decompensation (ascites, encephalopathy), ALBI (secondary)	Waiting-list mortality, transplant eligibility and prioritisation, perioperative mortality, early post-transplant survival
Hepatic Resection	Child–Pugh, ALBI, MELD, ICG-R15, ALICE grade, liver stiffness, HVPG, FLR volume/function	Post-hepatectomy liver failure (PHLF), perioperative morbidity and mortality, overall survival, recurrence-free survival
Percutaneous Ablation	Child–Pugh, ALBI, serum albumin, platelet count, AST/ALT, presence of portal hypertension	Overall survival, recurrence-free survival, risk of post-procedural liver decompensation
TACE	Child–Pugh, ALBI, PALBI, MELD, bilirubin, albumin, INR, presence of ascites	Overall survival, treatment tolerability, risk of post-TACE liver decompensation, ability to repeat TACE, transition to systemic therapy
TARE	Child–Pugh, ALBI, MELD, bilirubin, liver stiffness, dosimetry-adjusted functional reserve	Overall survival, radiological response, radiation-induced liver disease, hepatic toxicity
Systemic Therapy	Child–Pugh, ALBI, MELD, portal hypertension markers (platelets, liver stiffness), bilirubin	Overall survival, progression-free survival, treatment discontinuation, liver decompensation during therapy

MELD: Model for End-Stage Liver Disease; MELD-Na: Model for End-Stage Liver Disease Sodium; ALBI: albumin–bilirubin grade, ICG-R15: indocyanine green retention rate at 15 min; HVPG: hepatic venous pressure gradient; FLR: future liver remnant; AST: aspartate transaminase; ALT: alanine transaminase, TACE: Transarterial Chemoembolization; TARE: Transarterial Radioembolization.

**Table 2:** Systemic therapies for hepatocellular carcinoma and their impact on liver function.

Drug/Regimen	Therapeutic Class	Impact on Liver Function
Sorafenib	Multikinase TKI	May induce deterioration of liver function, particularly in patients with borderline hepatic reserve. Real-world data show higher rates of Child–Pugh worsening in CP-B patients. Hepatic decompensation often limits treatment duration.
Lenvatinib	Multikinase TKI	Comparable liver toxicity to sorafenib in CP-A patients; however, rapid tumour shrinkage may precipitate hepatic insufficiency in patients with limited reserve. Child–Pugh deterioration reported, especially in real-world cohorts.
Regorafenib	Multikinase TKI (second line)	Requires preserved liver function for eligibility. Liver function deterioration is common at progression and frequently precludes continuation beyond early cycles.
Cabozantinib	Multikinase TKI	Associated with liver function decline over time, mainly driven by cirrhosis progression rather than direct hepatotoxicity. CP-B patients show reduced tolerance and survival benefit.
Ramucirumab	Anti-VEGFR2 monoclonal antibody	Minimal direct hepatotoxicity; however, benefit restricted to patients with preserved liver function. Risk of ascites and portal hypertension-related complications reported.
Nivolumab	Immune checkpoint inhibitor (PD-1)	Generally well tolerated from a hepatic standpoint; immune-mediated hepatitis is uncommon but potentially severe. Liver function deterioration usually reflects cirrhosis progression rather than drug toxicity.
Pembrolizumab	Immune checkpoint inhibitor (PD-1)	Similar hepatic safety profile to nivolumab. Immune-related hepatitis requires prompt recognition and management; CP-B patients underrepresented in trials.
Atezolizumab + Bevacizumab	ICI + anti-VEGF	Overall favourable liver safety profile in CP-A patients. Bevacizumab may exacerbate portal hypertension and increase risk of variceal bleeding. Liver function deterioration at progression is a major determinant of outcome and access to subsequent therapy.
Durvalumab + Tremelimumab (STRIDE)	Dual immune checkpoint blockade	Limited direct hepatotoxicity; immune-mediated hepatitis more frequent than with monotherapy but manageable. Liver function preservation critical for sustained benefit.

TKI: tyrosine kinase inhibitors; ICI: Immune Checkpoint Inhibitors; VEGFR: Vascular Endothelial Growth Factor Receptor; PD-1: Programmed Death-1.

## Data Availability

No new data were created or analysed in this study.
